# Atlanta Academy of Medicine

**Published:** 1876-03

**Authors:** H. F. Scott

**Affiliations:** Reporter


### Atlanta Academy of Medicine.

H. F. SCOTT, M.D., Reporter.

Atlanta, January 18, 1876.

Academy met, Dr. Miller, President, presiding.

Dr. John M. Johnson read a paper on Opium Poisoning, in which he objected to the doctrine of the antidotal properties of certain drugs in opium poisoning, and pronounced the theory a myth.

On the conclusion of the Doctor's paper, Dr Todd remarked that the effects produced by the opium taken by his patients precludes the idea of cow dung as a constituent. Again, Dr. Johnson gives no reason for veratrum not being an antidote to opium.

Dr. Johnson said he only reported his own experience; opium and its preparations were in everybody's hands, and was regarded as a harmless drug; so much so that until lately no inquiry had been made into its components; at present knew of several constituent parts, but of a great many we know nothing. If this is the case, why speak of antidotes? He remarked that no man of reputation would stake himself on belladonna as being an antidote, yet it was a remedy very potent for evil; the same thing with veratrum. He used them hesitatingly and in small quantities. Remarked, also, that the discoverer of veratrum considered it deobstruent, sudorific, and especially sedative; then if it was wanted to keep up the circulation, would you give veratrum? Manifestly no. Under peculiar circumstances, as firm pulse and heat of skin, he would give it for the sedative effects; but where it was necessary to stimulate and send arterial blood to the brain, to keep up its energies, he would not give veratrum. He further remarked that he was seeking for truth, and if wrong, wished to be corrected, and merely made these suggestions in the interest of the profession.

Dr. Todd replied that he did not, of course, mean to convey the impression in his paper that veratrum was chemically an antidote to opium. The effects being different, he considered the drugs antagonistic. Thought poisoning was not the result of depression of the heart's action, but paralysis of the respiratory function and want of stimulation to the brain.

Dr. Johnson considered the respiratory act essential as a stimulus to cardiac action. If the blood is not oxygenated, the heart's action ceases immediately.

Dr. Todd asked if that is the case, why does not a man's heart cease to beat when he is choked from hanging? He asked Dr. Johnson how long did it beat when a man was hung.

Dr. Johnson replied, from ten to fifteen minutes; spoke officially.

Dr. W. F. Westmoreland wanted the Doctor to define the term antidote.

Dr. Johnson replied that he meant "*good against.*"

The President thought Dr. Todd's idea was not that they were chemically antagonistic to each other, but that the effects were different. Remarked, also, that Dr. Johnson censured veratrum and belladonna because they were both dangerous remedies. Thought his suggestions were of value to prohibit rash experiment. Concluded by asking Dr. Todd how much veratrum he thought could be safely given.

Dr. W. F. Westmoreland had seen a man take an ounce of veratrum, mistaking it, whilst tapering off from a spree, for paregoric. It produced most terrific vomiting and alarming prostration, cold extremities, cold sweats, etc; pulse 36. Arrived a half hour afterwards, and found him in above condition. Opium and stimulants were given happily. Thinks there is no doubt opium will counteract the effects of veratrum; he always gives it in vomiting from veratrum; he had never seen veratrum given for opium poisoning. Said he considered it an antidote to the effects of opium, but not in the sense Dr. Johnson would have it to act—chemically.

Dr. J. G. Westmoreland thought opium acted as a stimulant or excitant of the brain, and that the proper antidote would be found in the remedy acting as a sedative. He therefore thinks it reasonable that veratrum, being a powerful sedative, either directly or indirectly, to the brain, should prove a useful antidote for the poisonous effects of opium. Although he had used belladonna for this purpose, he thinks the veratrum treatment probably more rational.

Dr. J. M. Johnson asked, is opium a sedative? Is coma the effect of stimulation?

Dr. J. G. Westmoreland—No; it is a stimulant of the brain, but coma may be the result of destroyed function of the brain, from excessive excitement as well as sedation. Insensibility may be the result of anything that destroys the function of the brain. Too much blood, and too little in the brain, alike cause insensibility.

Dr. J. M. Johnson—The first effect of opium is that of stimulation, next drowsiness, and finally insensibility, or coma. Is this stimulation?

Dr. J. G. Westmoreland—No; it is not stimulation, but the result of over-stimulation. The function of the brain is deranged by excessive excitement. Even when the stimulation has not been sufficient to disturb the function, but only in amount to make it more perfect, a state of comparative inactivity succeeds the exhilaration. Like a wave of the sea, in proportion to the height it rises will be its descent below the normal standard.

Dr. J. M. Johnson—But if the man is already depressed, would you give veratrum?

Dr. J. G. Westmoreland—I would in order to change the state of the brain, which is the subject of paralyzing excitement, not the result of excitement which passed off hours ago.

Dr. Baird—What do you mean by stimulation of the brain?

Dr. J. G. Westmoreland—Increased excitement.

Dr. Baird—then you believe sleep to be a state of cerebral excitement?

Dr. J. G. Westmoreland—No; I never mentioned sleep in connection with the effects of opium.

Dr. Baird—Does not opium produce sleep?

Dr. J. G. Westmoreland—No; it produces excitement, exhilaration, and as the excitement subsides sleep may ensue. In one that has been suffering for some time from pain and loss of sleep in consequence, sleep may come on as the pain is relieved by opium notwithstanding, and in despite its exhilarating effect.

Dr. Miller was struck by the fact of the relief following the vomiting, which he didn't doubt was produced by the veratrum taken, but would not any emetic produce the same effect? Knew of forty-five drops having been taken by a physician, from which he became insensible. No vomiting, and died three hours afterwards. This case caused him to use caution in the administration of veratrum. He thought the question as to the antidotal properties of veratrum in opium poisoning was still *sub judice.*

Dr. Todd knew veratrum was a poison, and he had seen cases where the patients would have died if not for the use of opium and stimulants.

Dr. Miller called attention to the experiments of Matteuchi, showing that exosmosis and endosmosis going on was stopped if morphine was dissolved in one of them. Thought probably that veratrum might act in that way, preventing the absorption of morphia. He said that opium, by preventing endomosis and exosmosis, cured diarrhœa.

Dr. Todd entirely rejects this hypothesis; for if morphia prevented absorbtion, how does it get into the circulation itself? He thinks the astringent action of opium is referable to three phenomena: 1st, by partially paralyzing the peristaltic action of the intestine; 2d, by diminishing all of the secretions except that of the skin; 3d, by retarding tissue metamorphosis.

Dr. Todd, upon being asked in what way veratrum was antidotal to opium, and how he reconciled the known depressent effect of both remedies on the circulation of the heart, answered, that he was not any more able to explain how it antagonized opium than he was to elucidate how quinine cured ague, sulphur scabies, or mercury syphilis; that they do it we know, and give them without knowing their modus operandi. No more potent agent in shock from a severe injury, when the pulse is weak and the heart's action depressed, than opium, is at our disposal. We give it frequently to relieve congestion, although we know it depresses the heart's action; we treat many inflammations as if the pharynx, larynx, and buccal mucous membrane, by applying to them an agent which increases inflammation; we give a cathartic frequently to cure a diarrhœa.

Dr. Calhoun reported a case of supposed melanotic cancer of the cornea; certainly it was encephaloid in character. A lady thirty-one or thirty-two years old, six months ago was thrown from a horse, and when lifted up there was a little ecchymosis of right eye at junction of cornea and sclerotic. It increased in size, and was removed in Spartanburg, S. C. Afterwards was thrice removed; the last removal being about six weeks ago. When I saw her it was just protruding between the eyelids. The original seat of trouble was conjunctiva around the cornea. Intervals were shorter and growth more rapid after each removal. It presented three or four little melanotic points on cornea, which were very rare. It was easily broken down by probe, like brain substance. Remarked that the only remedy was removal of the eye-ball, which was done in the ordinary way. The danger is that the nerve was affected before the removal. Statistics of very few such cases are on record, and he would like to know the experience of members as to its return

Dr. W. F. Westmoreland's experience was that encephaloid cancer is generally rapid in growth, and tissues become early affected. Epithelioma is less malignant, and he has removed several without return. Was of the opinion that some cancers are local, and others not. Thought the above case was of local origin. Has never known encephaloid cancer to fail to return.

Dr. Rudicill (visiting) remarked that the report of proceedings of Academy, in the Journal, were always looked for with great interest by the profession. Said the Academy would confer additional favor on the profession, especially those in the country, by discussing such diseases as might be prevalent at any time.

Dr. Baird wished to call attention to the effects of belladonna in promoting contraction of the vesical sphincter. Has recently treated six cases of incontinence of urine—three in adults, three in children, and had used tr. of belladonna, in dose of fifteen drops, three or four times daily, in adults, with invariable success. In these cases he was unable to ascribe the trouble to any ascertainable cause.

Dr. W. F. Westmoreland thought this treatment was not generally known and not generally practiced, and would like Dr. Baird to prepare a paper stating the facts in each case and the quantity used.

Dr. Jno. M. Johnson has been accustomed to use this remedy for a long time, and thought everyone did the same. He seldom used any other remedy for the condition. Its history shows it has been used for this affection many years. Related several cases in which he used English extract of belladonna with good results.

Dr. Todd remarked that the known variability of strength of the tincture and extract, leads him to inquire if atropia has been used, and the result.

Dr. Baird called attention to the importance of continuing its administration several days after the desired effect had been secured, in order to effect a cure, and sometimes it was even necessary to secure the physiological effects.

The President thought the remedy acted by relieving the irritability at the neck of the bladder.

Dr. John M. Johnson said it was perfectly safe to use it when there was incontinence onor excessive repeated or constantly recurring flow of urine. Gives it in small quantities and watches its effects. If used long enough it would set up irritation in some of the urinary passages or bowels, and increase existing irritation of the lungs, as in consumption, where it should be given no longer than absolutely necessary. In some cases, as Bright's disease, or active inflammation and discharge of blood from the bladder, it does not coırect the trouble.

Academy adjourned.

---

Atlanta, February 1, 1876.

Academy met, the President, Dr. Miller, in the chair.

Dr. Scott reported a case of poisoning of a family of five persons. Five out of a family of eight had eaten some souse meat for breakfast. Soon afterwards those who had partaken of it commenced vomiting and purging; one of them, the father, seemed to have vomited all the poison, as he was not affected afterwards, and only required a little whisky to quiet his stomach. The others continued to vomit and purge during the whole day, becoming gradually weaker and weaker, until about 5 P.M., when he was sent for. On arriving he found them in an alarming state of prostration, suffering great pain in the bowels, and out of their heads, making it difficult to control them. The two youngest, especially, were almost lifeless. He immediately gave hypodermic morphia, repeated every half hour for three times. Also gave sulphate of zinc and sent for stimulants and mustard. The morphia produced quick relief, quieting the patients and relieving the head symptoms. The zinc acted happily, producing in half an hour copious vomiting from each.

In the meantime, Drs. W. F. Westmoreland and Thomas M. McIntosh arrived, at his request, when the treatment was continued, with the addition of milk punch in large quantities, and mustard freely to extremities. He remained with them until 1 o'clock. Patients continued to improve, and finally went off into a quiet sleep, from which they awoke at 8 o'clock next day. All did well afterward; one had dysentery, which yielded kindly to treatment. It will be noticed that only those in the family who partook of the meat were affected. Again, a cat eat some of the meat, and vomited freely. It was then a reasonable conclusion that the meat was at fault. The attending physician concluded that the souse meat had been pressed in copper vessels, though this was emphatically denied by the person from whom it had been obtained. The family said that they scraped off a mould from the souse before eating it.

Dr. Scott remarked, also, that there were worms vomited by one of the children and by the cat; these were of the lumbricoid character, and came away in detachments. How this was produced he knew not.

On the conclusion of the report, there ensued a discussion on the power of certain remedies to disintegrate worms.

Dr. Miller thought there was no evidence to sustain the assertion made by some, and commonly believed by the masses, that certain medicines had this power. He has never met with a case, and believes it to be impossible.

Dr. John G. Westmoreland held the same opinion.

Dr. Todd remarked that probably the worms, in this case, had been bitten into pieces by the patients.

Dr. John M. Johnson thought medicines were capable of causing them to pass in detachments. When we give a remedy, it immediately affects and destroys the worm; in a very short time it becomes decomposed and passes away in detachments. Remarked that he had had some experience in poisoning from food. Related a case of poisoning in a boarding-house, from eating chicken salad. There was nothing possibly poisonous in the ingredients. He explained the case by saying that the salad was partially cooked, and the protenic compounds, under the influence of heat, having been placed on a shelf in a warm room, became poisonous. He would explain the above case in the same way, and thinks the fact of there being a mould goes far to substantiate his theory.

Dr. W. F. Westmoreland doubts the theory of worms becoming so soon decomposed. Has seen them thirty-six hours after being voided still alive. Thinks it more plausible they were bitten.

Dr. Taliaferro affirms that remedies do not macerate worms. He has found, as a most admirable remedy for worms, hyposulphite of soda, in doses of five, ten, or fifteen grains, in solution. This kills the worms absolutely. He is in the habit of not giving anything to move the bowels afterwards, as the remedy itself acts.

Dr. Rauschenberg has never met with worms cut up, and doesn't believe they ever are. He cannot imagine how it is possible for them to be so.

In reference to partially decomposed meats being poisonous, Dr. W. F. Westmoreland remarked that that was the kind that suited the epicure.

Dr. John M. Johnson, in reply, said that if decomposition sets in and the gases are allowed to escape, you have no poison; but if the gases are pent up, then there is a poison.

Dr. J. T. Johnson stated that some meats will not produce this poison readily, while others will, as the lighter meats, oysters for instance, not fresh, produce symptoms of poisoning. He referred to cases reported by Dr. Baird at last meeting of Academy, in reference to belladonna in promoting contraction of vesical sphincter. He believes that the belladonna acted, not by producing contraction or dilatation, but by relieving irritation. Remarked that Dr. Stanford, of Columbus, reported a series of cases to the Georgia Medical Association, in which hyperæsthesia of the neck of the bladder, and the result of it, were cured by introducing steel sounds.

Dr. J. T. Johnson reported a case of pruritus ani, which has annoyed him considerably. Stout, healthy young man, twenty-five years old, single, regular habits and temperate. The disease has lasted for months, and is very annoying to him night and day. It is altogether without the rectum, having commenced at first midway between the scrotum and rectum. He has examined the case carefully and repeatedly. The bowels are regular; no ascarides; liver all right; no disease of rectum. He has used remedy after remedy with no avail. He cauterized the rectum with a strong solution of nitrate of silver in glycerine, the idea being suggested from having cured a very intractable case of chronic dysentery with it. Probably there might be a tapeworm. The sphincter ani muscle is very powerful, and he now thinks of rupturing it as a means of relief.

Dr. Miller proposed that he make use of the anal pessary.

Dr. John M. Johnson once had a case of hemorrhoids, accompanied with violent itching. He treated her repeatedly with various remedies, and finally used the anal pessary, with no recurrence. Remarked that it could be worn with no inconvenience, save the escape of offensive gases.

Dr. J. G. Westmoreland said that Dr. J. T. Johnson's case was probably one of disease of the skin around the sphincter, and that the remedy should be applied to the skin. He recently had a case of excessive itching around the anus and extending into the anus somewhat, which he cured in a few days by applying once daily a mixture of glycerine, carbolic acid, and iodine.

Dr. J. T. Johnson remarked that the pruritus is frequently some distance from the seat of the cause giving rise to it, as at the termination of the mucous canal.

Dr. Taliaferro thinks this idea is the correct one; he thinks the case is suffering from gastric irritation, manifesting itself near the anus. Said the trouble may be brought about by the excessive use of tobacco or alcoholic stimulants, in a subject where there is a want of nervous tone, as the result of protracted nervous irritability. Said the trouble is rarely local; rarely will local remedies relieve it.

Dr. Rauschenberg is inclined to think it exists as an independent local disease. Has seen cases that were perfectly healthy, and no cause discoverable, and still there was pruritus. Thinks this case is not produced by distant irritation, but by local irritation of some kind. Thinks it is a perversion of nervous action at this point. Cured one case by solution of corrosive sublimate, followed by powdered camphor and calomel, equal parts; having received the idea from a source unknown at present.

Academy adjourned.

